# From Germ Cells to Implantation: The Role of Extracellular Vesicles

**DOI:** 10.3390/jdb12030022

**Published:** 2024-08-23

**Authors:** Anna Fazzio, Angela Caponnetto, Carmen Ferrara, Michele Purrello, Cinzia Di Pietro, Rosalia Battaglia

**Affiliations:** 1 Department of Biomedical and Biotechnological Sciences, Section of Biology and Genetics “G. Sichel”, University of Catania, 95123 Catania, Italy; anna.fazzio@phd.unict.it (A.F.); angela.caponnetto@unict.it (A.C.); carmen.ferrara@phd.unict.it (C.F.); purrello@unict.it (M.P.); rosalia.battaglia@unict.it (R.B.); 2 Department of Physics and Astronomy “Ettore Majorana”, University of Catania, 95123 Catania, Italy

**Keywords:** extracellular vesicles, exosomes, implantation, embryo, gametogenesis, germ cells, microRNAs, biomarkers, blastocoel fluid

## Abstract

Extracellular vesicles represent a large heterogeneous class of near and long-distance intercellular communication mediators, released by both prokaryotic and eukaryotic cells. Specifically, the scientific community has shown growing interest in exosomes, which are nano-sized vesicles with an endosomal origin. Not so long ago, the physiological goal of exosome generation was largely unknown and required more investigation; at first, it was hypothesized that exosomes are able to remove excess, reject and unnecessary constituents from cells to preserve cellular homeostasis. However, thanks to recent studies, the central role of exosomes in regulating cellular communication has emerged. Exosomes act as vectors in cell–cell signaling by their cargo, proteins, lipids, and nucleic acids, and influence physiological and pathological processes. The findings on exosomes are widespread in a large spectrum of biomedical applications from diagnosis and prognosis to therapies. In this review, we describe exosome biogenesis and the current methods for their isolation and characterization, emphasizing the role of their cargo in female reproductive processes, from gametogenesis to implantation, and the potential involvement in human female disorders.

## 1. Introduction

Intercellular communication is mediated by an extensive variety of signaling processes, and it is indispensable for the maintenance of homeostasis and the safety of the whole organism [1,2]. Traditionally, cell-to-cell communication involved direct interactions (gap junctions); autocrine, paracrine and endocrine mechanisms; and signaling molecules targeting cellular receptors [3]. Over the last few years, new mediators of intercellular communication in both prokaryotic and eukaryotic domains [3] have emerged: the extracellular vesicles (EVs). EVs have become a central hub in cellular biology, acting as mediators among cells not only in physiological but also in pathological conditions [4,5,6,7,8]. EVs include a heterogeneous class of vesicles delimited by a phospholipid bilayer [9]. They are constitutively released by numerous cell types in response to specific stimuli or cellular stress factors [4,10] and transfer their contents to the target cells. Currently, several subgroups of EVs have been defined, such as small EVs, large EVs, ectosomes, extracellular particles, and exosome-like vesicles [3]. However, due to the difficulties in isolation methods and the different classification criteria, three major groups of vesicles are essentially considered. Based on their size, density, morphology, biogenesis and surface markers, they can be classified in microvesicles (MVs), apoptotic bodies and exosomes. MVs, also known as microparticles, are released by budding or shedding of the plasma membrane [11] and show a diameter > 100 nm. The apoptotic bodies, with the broadest range of diameters (50–5000 nm), are produced by cells undergoing apoptosis [12]. Exosomes, nano-sized vesicles (from 30 to 100 nm in diameter), show a characteristic cup-shaped morphology bounded by a bilayer membrane and are secreted by most cells both in physiological and pathological conditions [13]. The biogenesis of exosomes mainly spans across three different stages. First, the cytoplasmic membrane invaginates to form endosomes, which in turn fuse to form early endosomes (ESEs). Then, ESEs invaginate again to encapsulate the intracellular material, forming multiple small intraluminal vesicles that are further transformed into late endosomes (LSEs), which later become multivesicular bodies (MVBs). Finally, MVBs fuse with the cytoplasmic membrane, releasing intraluminal vesicles (ILVs) into the extracellular environment [14,15]. Nowadays, accumulating evidence suggests that these secretory vesicles are released by all types of eukaryotic cells and are widely available in almost all bodily fluids, including urine, blood, cerebrospinal fluid, saliva, breast milk, seminal fluid and follicular fluid [16,17]. Precisely, they can function as intercellular transmitters to deliver the cargo molecules necessary for cell-to-cell communication via interaction on the cell surface, taking part in many biological processes [2]. Their intercellular trafficking includes proteins (such as annexins, tetraspanin, integrin family, proteoglycans, lectins, and proteins involved in apoptosis) [18], lipids (such as cholesterol, diglycerides, sphingolipids, phospholipids, prostaglandins, and leukotrienes) [19] and nucleic acids (DNA, mRNAs, and non-coding RNAs) [20]. The term exosome was coined for the first time by Johnstone et al. in 1987 [21], representing the smallest unit of EVs delivered to the extracellular space [22]. Exosomes are usually hemispherical, circular, and elliptical cups with a concave side [23]. Despite notable progress, the characterization and quantification of exosomes remain considerable due to the complexity and lack of standard isolation methods [24,25]. Today, a set of standardized methods for exosome isolation and characterization have been widely accepted. In order to ensure reproducibility and comparability across the different studies and define stringent classification parameters, the International Society for Extracellular Vesicles (ISEV) proposed the Minimal Information for Studies of Extracellular Vesicles (MISEV) guidelines for the field in 2018. The MISEV 2018 guidelines include tables and outlines of suggested protocols and steps to follow to document specific EV-associated functional activities [26,27]. The most commonly used methods in exosome isolation are ultrafast-centrifugation, density gradients, filtration (by using filters with pore diameters of 0.8, 0.45, 0.22, and 0.1 μm) [28] and exosome extraction kits based on polyethylene glycol (PEG) precipitation [28]. While scanning and transmission electron microscopy (SEM and TEM) have been used to observe cup shape, confocal microscopy is employed for live imaging of exosomes uptake and their intracellular mobility [29]. Nanoparticle tracking analysis (NTA) and dynamic light scattering (DLS) are also used to measure particle distribution, concentration and sizes in the range of 30–150 nm [30]. Western blotting is commonly applied to detect the presence of molecular markers inside and outside exosomes. Nowadays, the international standard is the detection of three positive proteins (such as transmembrane Tetraspanins CD63, CD9 and/or CD81, soluble protein TSG101 and cytoplasmic protein Alix) and one negative protein (such as endoplasmic reticulum protein Calnexin and nuclear proteins histone and Golgi protein GM130) [27,31,32]. In 2019, Zhang and Lyden optimized asymmetric flowfield flow fractionation (AF4) technology for separating and characterizing extracellular nanoparticles, particularly exosomes [33]. However, one of the main limitations of AF4 is that it fractionates samples based on their size; thus, particles with the same hydrodynamic size but with different morphologies, surface molecules and other biophysical properties cannot be separated from each other via AF4 alone [33]. In 2021, a paper published in the *International Journal of Nanomedicine* [25] proposed a silicon nanowire (Si NW) label-free luminescent sensing system. It is functionalized with a specific antibody able to selectively bind the vesicles with CD81+ antigen. Tested with follicular fluid and blastocoel samples, Si NW label-free luminescent sensors showed high selectivity and sensitivity, outperforming standard mass approaches like ELISA. The functionalization processes used for these sensors make them suitable for commercial applications [25]. Initially, exosomes were underestimated as vesicles for cellular waste disposal; however, they are now recognized as crucial contributors in intercellular communication [34,35]. Exosomes are able to release their contents into the cytosol of acceptor cells by using their surface molecules’ receptor–ligand interaction, endocytosis, or direct fusion with the targeting cell membrane [36]. Their bioactive cargoes, up-taken by recipient cells through different mechanisms, can influence molecular pathways and cellular function [37,38,39]. In, 2007 Valadi et al. demonstrated for the first time that, in addition to DNA, mRNAs, proteins and lipids, exosomes can transport non-coding RNAs (ncRNAs); in particular, the authors revealed the presence of microRNAs (miRNAs) in exosome cargo, which could be delivered to other cells, influencing their functions and gene expression. This represents a novel mechanism of genetic exchange between cells, which they proposed to call “exosomal shuttle RNA” (esRNA) [40]. Research is ongoing to better understand the sorting mechanisms and practical impacts of ncRNAs in exosomes: technological advancements in high-throughput sequencing and bioinformatic analyses are aiding in the detailed characterization of exosomal ncRNAs, emphasizing the complexity of intercellular communication and their potential for innovative diagnostic and therapeutic strategies [41]. These discoveries suggest that the investigation on circulating exosomes and their cargo could provide new opportunities for liquid biopsy in precision medicine, highlighting the potential role of exosomes as biomarkers for cancer diagnosis and prognosis prediction [41,42,43]. Exosomes showed greater advantages in liquid biopsy: in most cases, their abundance in biofluids could contribute to easier isolation of vesicles [39]; exosomes are secreted by living cells and contain several forms of biological information from their origin cells [37,39]; they are innately stable thanks to their lipid bilayers and thus stably circulate both in physiological conditions and in the harsh tumoral microenvironment [42]. However, one of the most significant limits for the application of exosomes in liquid biopsy is isolation with high efficiency and purity, due to their nanoscale size and intrinsic heterogeneity [44,45,46]. Ultrasensitive and precise detection is necessary for the development of exosome-based cancer diagnostics, since they represent only a small part of all exosomes in bodily fluids [42]. The great predictive potential of exosomal cargo finds application not only in cancer but also in reproductive medicine, pointing out the possibility of using the molecules transported inside exosomes as possible markers of gametes, embryo and implantation quality [47].

## 2. Exploring the Activity of Exosomes in Gametogenesis and Fertilization

### 2.1. Spermatogenesis

**Figure 1:**
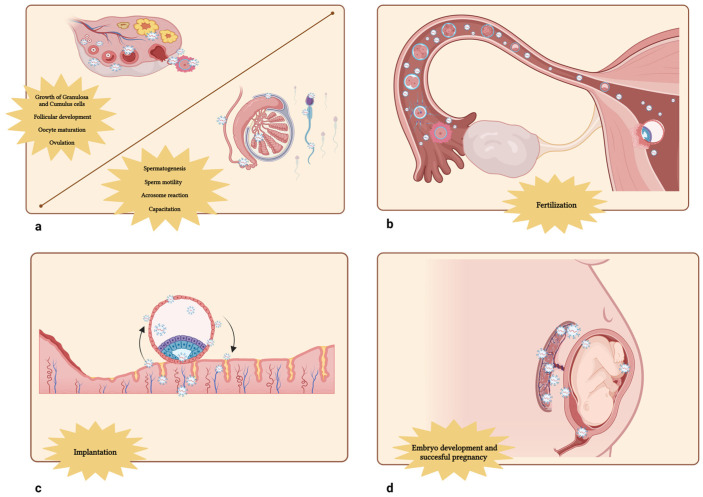
The molecular actions of EVs in gametogenesis [14,23,49,53,54,56,57,58,59,65,67,68,69,71,72,73,74,75,76,77,78,79,80,81,82,83] (**a**), fertilization [48,84,85,86,87] (**b**), implantation [88,89,90,91,92,93,94,95,96,97,98,99,100,101,102,103,104,105,106,107] (**c**) and embryo development [108,109,110,111,112,113,114,115,116,117,118,119,120,121,122,123,124,125,126,127,128,129,130,131,132,133,134,135,136,137,138,139,140,141,142] (**d**) processes. Edited using Biorender https://www.biorender.com/ (accessed on 8 August 2024).

**Figure 2:**
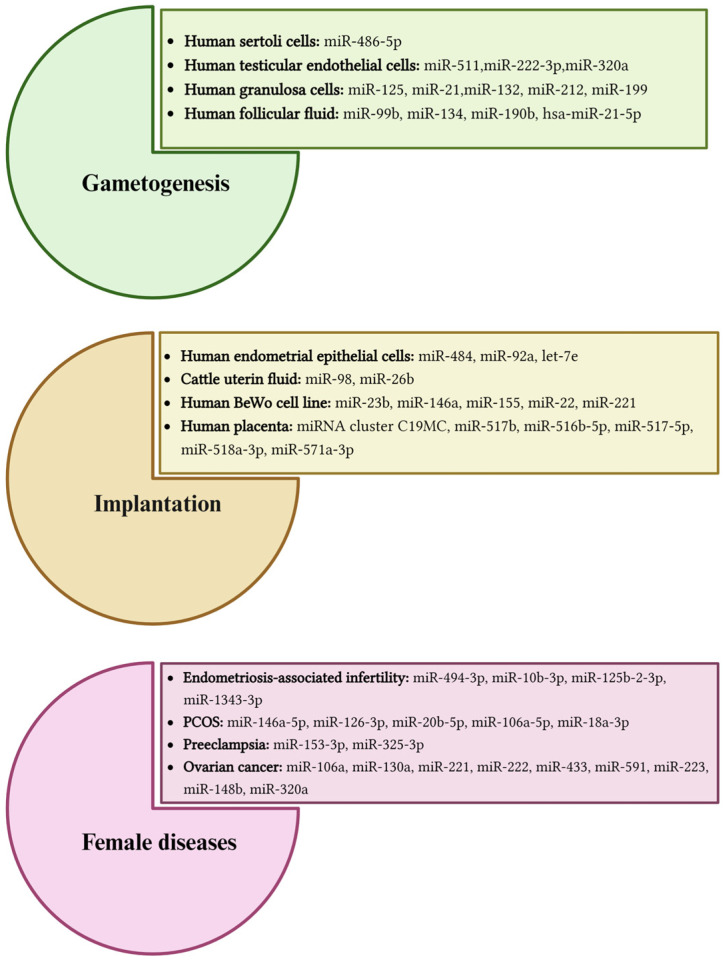
MicroRNA cargo of extracellular vesicles involved in gametogenesis [14,23,49,68,70,83], implantation [22,101,102,117,118,119,120,122,124,125,126,127,128,129,130] and female diseases [143,144,145,146,147,148,149,150,151,152,153,154]. Edited using Biorender https://www.biorender.com/ (accessed on 8 August 2024).

In sexual reproduction, sperms and oocytes represent the fundamental protagonists generating a new organism. The maturation and functionalities of germinal cells are critical points for reproductive success [48]. In recent years, scientific evidence suggests that exosomes play significant roles in gametogenesis, facilitating communication between germ cells and the somatic cells and influencing the proliferation, differentiation, and maturation of the gametes [48,49,50,51,52] (Figure 1). In spermatogenesis, the sperm maturation process and the acquisition of fertilization capability involve a series of intricate interactions along the male reproductive tract [53,54]. The Sertoli cells support and coordinate germ cell development and spermatogenesis by offering nutrients and participating in the formation of the blood–testis barrier [55]. A few studies reveal that Sertoli cells are able to release exosomes in the testis and allow sperm differentiation into the spermatogenic tubules [48,56,57]. The epididymis, comprising the caput, corpus, and cauda, facilitates sperm maturation, transport, and storage: each segment offers a unique habitat optimized for different stages of sperm development [58,59]. Sperm undergoes crucial changes in morphology and function during its transit through the epididymis, where it interacts with different microenvironments. Throughout this transit, sperm modifies membrane composition and surface proteins, influenced by interactions with the surrounding intraluminal fluid. Numerous studies have demonstrated the secretion of EVs by epididymal epithelial cells (epididymosomes) and the prostate (prostasomes) [60,61,62,63,64]. These vesicles are able to orchestrate sperm pathways—such as capacitation, acrosome reaction, protection against oxidative stress, stability and fluidity—by transporting bioactive molecules such as sperm adhesion molecule 1 (SPAM1), glioma pathogenesis-related 1-like protein 1 (GliPr1L1), metalloprotease, proto-oncogene tyrosine-protein kinase Src (cSrc), macrophage migration inhibitory factor (MIF), dicarbonyl/L-xylulose reductase (DCXR), Liprin α3,and also miRNAs [49,65,66,67]. In fact, it seems that exosome-derived miRNAs are a new component of cell–cell communication: a recent study revealed that Sertoli cell exosome-derived miR-486-5p is able to down-regulate *PTEN* and up-regulate the expression of *Stra8*, promoting the differentiation of spermatogonial stem cells (SSCs) into spermatogonia in mice [23,68]. A study conducted in 2022 [69] investigated and analyzed the protein and miRNA components of human testicular endothelial-cell-derived exosomes (HTEC-Exos), revealing that the deregulation of the expression of miRNAs (hsa-miR-511, hsa-miR-222-3p, and hsa-miR-320a) in sperm cells, epididymis, seminal plasma, and EVs (i.e., exosomes and microvesicles) may lead to alterations to spermatogenesis and various forms of infertility [70] (Figure 2).

### 2.2. Oogenesis

In mammalian female gametogenesis, mature ovarian follicles, comprising oocytes, somatic cells (cumulus granulosa, mural granulosa and theca cells), and follicular fluid (FF), represent a critical dynamic microenvironment rich in metabolites and hormones to ensure follicular development and oocyte maturation [71]. Bidirectional communication between gametes and granulosa cells in the follicle occurs either directly by a network of gap junctions or through paracrine, autocrine and endocrine signaling factors in the FF [72,73]. These interactions guide the modulation of oocyte transcriptional activity to produce a competent oocyte able to support fertilization and preimplantation embryo development [74,75,76,77]. Emerging evidence highlights that exosomes contribute to this intricate interplay by transporting miRNAs that regulate several pathways involved in folliculogenesis [78], including WNT, TGFβ, MAPK, ErbB, and ubiquitin-mediated signaling pathways [49,79,80]. Many studies on the involvement of EVs from FF in follicular maturation use animal models. Hung et al. [81] have demonstrated that bovine follicular EVs are up-taken by bovine or mouse cumulus cells, consequently inducing the expansion of cumulus–oocyte complex (COC) thanks to the modulation of Prostaglandin-endoperoxide synthase 2 (*Ptgs2*) and Pentraxin 3 (*Ptx3*) expression, genes known to be involved in this process [81]. EVs originating in early, mid-estrus and pre-ovulation stages of oocyte maturation in mares present dissimilar concentrations and different miRNA contents depending on the stage. In particular, miR-125 and miR-199, probably secreted by granulosa cells and packaged in EVs, were particularly expressed in the pre-ovulatory stages, being involved in oocyte maturation and cumulus proliferation. MiR-21, miR-132, and miR-212, whose expression seems to be hCG/LH-related [14], regulate gene expression, inducing granulosa cell proliferation and the maturation of the cumulus–oocyte complex [82]. In 2014, Diez-Fraile et al. [83] identified four different expressed miRNAs in EVs from FF comparing younger with older women: specifically, they found miR-99b, miR-134 and miR-190b to be upregulated and miR-21-5p to be downregulated in older women [14]. These miRNAs regulate genes involved in heparan-sulphate proteoglycan expression, carbohydrate digestion and absorption, and apoptosis, and their altered regulation could affect follicle development and oocyte maturation [14,49,83] (Figure 2).

### 2.3. Fertilization

Finally, it seems that exosomes may also take part in the fertilization process, which represents a complex regulatory mechanism involving sequential events essential for the successful merging of sperm and oocytes [49] (Figure 1). When sperm passes through the uterus, the uterine-cell-derived exosomes (uterosomes) carry transmembrane proteins and glycosyl phosphatidylinositol junction protein (SPAM1), which are essential for sperm fertilization and enhance the ability to cross the cumulus cell [84]. Similarly, when sperm passes through the oviduct, oocyte-derived exosomes (Oc-Exo) deliver the sugar protein membrane Ca2+-ATpase4 (PMCA4) to the sperm surface, improving the resistance to zona pellucida hydrolysis and reducing multiple sperm fertilization [48]. Moreover, Oc-Exo promote spermatozoa motility and their capacitation, thanks to the induction of tyrosine phosphorylation and consequently activating the acrosome reaction [85]: in particular, Oc-Exo carries two tetraspanins—CD81 and CD9 [48]—involved in sperm–egg fusion, acting independently of each other [48,86]. Other molecules participate in the process of sperm–egg fusion, such as glutathione peroxidase-5 (GPX5), SPAM1, prostate-specific antigen (PSA), kinesin family member 5B (KIF5B), annexin A2 (ANXA2) and kallikrein 2 (KLK2), which are delivered by human-semen-derived exosomes [87]. Thanks to this finely regulated communication among gametes, orchestrated by a variety of factors and signaling molecules, the development and implantation of the embryo will be able to proceed.

## 3. Exosomes and Embryo Implantation

### 3.1. EVs as Actors of Endometrial Receptivity and Successful Implantation

Embryo implantation is a critical step and requires a close dialogue between the embryo and maternal tissues coordinated by molecular and physiological signaling networks [88]. In humans, the regular dialogue between the embryo and endometrium can only occur during a short period called the “window of implantation” (WOI) [89], extended from the 20th to the 24th day of the menstrual cycle [90]. It represents a receptive phase characterized by several morphological and molecular modifications, predisposing the endometrium to successfully accept, protect and develop the embryo [91,92]. Perturbations of this process provoke implantation failure, accounting for approximately 75% of human pregnancy losses [93,94,95,96]. During the WOI stage, the uterine cavity undergoes cyclic changes regulated by hormones (estrogen, human chorionic gonadotropin (hCG) and progesterone) [97,98] and molecules such as cytokines (e.g., IL6), growth factors (e.g., IGF-2), chemokines (e.g., CX3CL1) and adhesion molecules (e.g., integrins like αvβ3) [96]. However, the involvement of EVs in cell-to-cell interactions during embryo–maternal crosstalk has recently been investigated [99,100] (Figure 1). Data in the literature reveal that both endometrial epithelial and stromal cells are able to secrete EVs: in 2013, for the first time, Ng et al. [101] characterized the miRNA cargoes of EVs released by endometrial epithelial cell line ECC1. The authors identified hsa-miR-484, hsa-miR-92a and hsa-let-7e, whose target genes explain their actions on important pathways for implantation, like adherens junctions, ECM–receptor interaction, Jak-STAT, and VEGF-signaling pathways [101,102]. Similarly, in a study conducted by Greening et al. [103], the authors identified unique protein cargoes of human endometrial epithelial exosomes, like ADAMTS15, HSPG2, and EGFR, which perform a key role in implantation-related pathways [102]. The cargo is hormonally regulated in accordance with the phases of the menstrual cycle. Moreover, exosomes are up-taken by human trophoblast cells, altering their properties and enhancing their adhesion capability at implantation by the active focal adhesion kinase (FAK) signaling pathway [100,103,104]. Even human endometrial stromal cells are able to secrete EVs, e.g., by the hypoxia-inducible transcription factor HIF2α and its downstream target *Rab27b*, which controls vesicular trafficking [105]. These EVs are adsorbed by stromal, endothelial, and trophoblast cells in the uterine microenvironment, modulating their functions [102,105]. Liu et al. [106] discovered that endometrial stromal EVs are absorbed by trophoblast cells, enhancing their invasiveness: this is determined by the upregulation of N-Cadherin expression in the trophoblast cells, due to high levels of *SMAD2/3* in the trophoblasts in response to EVs [102,106]. Therefore, EVs are considered crucial players during the mechanisms of implantation and placentation, supporting decidualization, blood vessel formation and trophoblast development processes [102,107].

### 3.2. The Immune-Modulatory Effect of EVs on Pregnancy

Embryo adhesion to a receptive endometrium requires the protection of the embryo from the maternal immune system before implantation, but also the maternal endometrial inflammatory response after implantation promoting embryo invasion [108,109,110] (Figure 1). During embryo attachment, embryonic EVs seem to transport molecules potentially able to modulate the host endometrial immune system [111,112]. An increasing repertoire of uterine CD56, CD16, natural killer (uNK) cells, T lymphocytes, B cells, and dendritic cells are recruited at the implantation site during embryo implantation due to their ability to secrete immunosuppressive IL-10 [113,114] to protect the embryo from maternal immune attacks. Moreover, this modulation seems to depend on miRNA and protein EV cargo [115] being able to influence the establishment of pregnancy, fetal development and survival during pregnancy [116,117]. Moreover, there is increasing evidence that uterine fluid EVs (UF-EVs) have potent effects on the maternal immune system during implantation. Nakamura et al., in two papers published in 2019 and 2021 [118,119], reported that in cattle, during receptivity, UF-derived EVs carry miRNAs, bta-miR-98 [118] and bta-miR-26b [119], which target and negatively regulate immunoregulatory genes in endometrial recipient cells (including *CTSC*, *IL6*, *CASP4*, and *IKBKE*, and *PSMC6*, *CD40*, and *IER3*, respectively) [120]. Gene set enrichment analyses of downstream target genes highlighted the involvement of neutrophil activation in immune response and neutrophil-mediated immunity as potential immunomodulatory functions [120]. Embryos are able to release some immunosuppressive molecules (such as hCG and HLAG) inside EVs, inducing the production of immunosuppressive factors (e.g., IL-10 [121]) to protect themselves from maternal immune responses during the implantation process. For instance, earlier evidence suggests that mouse embryonic EVs containing progesterone-induced-blocking factor 1 (PIBF) could interact with CD4+ and CD8+ peripheral T-cells, causing IL-10 production and immunosuppression [121]. Similarly, in a study published in 2019 [122], the authors suggested that HSPE1-associated trophoblast cell line BeWo can release EVs that are able to suppress regulatory T-cell (Treg) signaling by modulation of miRNAs (like hsa-miR-23b, hsamiR-146a, hsa-miR-155, hsa-miR-22, and hsa-miR-221) and protein (HSPE1) expression on the T-cell surface and lumen, highlighting their potential to regulate immune cell function during embryo implantation [120]. Another study conducted by Rai et al. in 2021 [123] highlights that EVs present in human UF contain antioxidative regulators (*SOD1*, *GSTO1*, *MPO*, and *CAT*) specifically during the secretory phase [123]: they are able to reduce ROS levels, facilitating embryo apposition and its attachment to the endometrium [120]. The control and regulation of the communication between the mother and developing embryo also require the contribution of the placenta. It is an essential organ performing vital functions (ensuring gas exchange, nutrient and waste transfer, immunoglobulin transport, and hormone secretion [22] for the fetus to support its growth and survival and maintain the pregnancy. Crosstalk between the fetus and mother can involve the simple diffusion of molecules through tissue layers but also the extracellular vesicles, especially exosomes [117]. During pregnancy, various types of placental cells are able to secrete exosomes—placenta-derived exosomes—whose concentration continuously increases in maternal circulation over the first trimester of pregnancy [48]. They may be differentiated from other exosomes by the presence of placenta-specific growth factors, DNA fragments, mRNAs and miRNAs, which are involved in regulating the physiological function of the maternal uterus and fetal development [48]. Among the most interesting miRNAs, we can find several miRNAs located in chromosome 19—miRNA cluster C19MC—which is the largest cluster of miRNAs in the human genome [124]. MiRNAs within the human C19MC include 46 miRNAs that are expressed during pregnancy only in the placenta, the so-called placenta-associated miRNAs [125,126], secreted into the maternal circulation by exosomes, where they function in placental–maternal signaling [127,128]. For example, exosomal miR-517b increased the expression of *TNFα* and/or other death ligands [117]; instead miR-516b-5p, miR-517-5p, and miR-518a-3p are shown to influence the PI3K-Akt and therefore the insulin signaling pathways, and their expression levels seem to be regulated by various stimuli, including oxidative stress and glucose levels [22,129]. Furthermore, the exosomal transfer of placenta-specific miR-571a-3p into NK cells repressed cGMP-dependent protein kinase 1, a crucial mediator of nitric oxide signaling [117,130] (Figure 2).

### 3.3. Embryo-Derived EVs

Different papers underline the EV cargo as able to modulate embryo–mother bidirectional crosstalk [131,132] (Figure 1). In vivo, EVs have been found in uterine fluid and, in vitro, in the culture medium, released from endometrial cancer cell lines (ECC1) [101,133]. Even human embryos, obtained from in vitro fertilization cycles (IVF), can secrete exosomes and microvesicles in the culture medium, and it has been demonstrated that embryo EVs are received by primary endometrial cells [134]. Therefore, accurate characterization of their cargo could provide useful information about the quality of the embryo and endometrial receptivity in assisted reproductive medicine; actually, one of the most fascinating challenges in reproductive medicine is the identification of new biomarkers able to indicate the best-quality embryos to implant [14]. Currently, preimplantation genetic screening (PGS) is often associated with the morphological evaluation of embryo quality to detect euploid embryos to implant. Despite the potential of PGS, the invasive removal of cells from the trophectoderm is still considered a critical procedure [135]. For these reasons, spent culture medium analysis could represent an innovative method to assess embryo competence during implantation [14]. Giacomini et al., in 2017, demonstrated for the first time that human preimplantation embryos at different developmental stages (at day 3 and day 5 after fertilization) can release EVs during their in vitro culture for ART (assisted reproductive technologies) [136]. The authors isolated and characterized the exosomes from embryo-derived conditioned media, according to the guidelines of the MISEV; moreover, the embryo vesicles can be internalized by cultured endometrial cells, suggesting their ability to communicate with the maternal side [136]. Furthermore, co-culture techniques using both embryonic and endometrial cells have been introduced in a paper conducted by Bhadarka et al. [137], with the intent to mimic the uterine microenvironment and improve the quality of the embryo; the authors obtained better quality blastocysts with a higher implantation rate by culturing human embryos coming from women who had undergone intra cytoplasmic sperm injection (ICSI) with cumulus cells [14,137]. Investigating the nucleic acid constituent of the EVs released by the embryo in the culture media, in 2017, Pallinger et al. demonstrated, by using flow cytometry, that only embryos releasing a low number of vesicles were competent, probably because a higher presence of nucleic acid could be related to cell injury and, consequently, embryo damage [138]. Other studies have also shown the presence of miRNAs in the embryo culture medium, correlated with in vitro fertilization methods, embryo aneuploidy and pregnancy outcome [139,140]; Abu-Halima et al., by analyzing extracellular vesicle secretion and miRNA expression in the spent culture media after embryo transfer, observed a reduced miRNA amount related to decreased EV secretion from an embryo successfully implanted in comparison with an embryo with a negative outcome [14,141]. Another approach to evaluate embryo quality is revealed in a study published in 2019 by Battaglia et al. [142]: for the first time, the authors demonstrated the human embryo’s ability to secrete exosomes enriched with miRNAs inside the human blastocoel fluid (BF). Bioinformatic and comparative analyses identified the biological function of these miRNAs in critical signaling pathways controlling embryo development, such as pluripotency, cell reprogramming, epigenetic modifications, intercellular communication, cell adhesion and cell fate. MiRNAs of BF reflect the miRNome of embryonic cells and their presence in exosomes, strongly suggesting their important role in mediating cellular signaling among blastocyst cells. Their characterization is important for studying the earliest stages of embryogenesis and the complex paths regulating pluripotency. Above all, miRNA expression profiles in BF could be used as possible minimally invasive biomarkers of embryos, predicting the implantation rate in IVF cycles [14,142].

## 4. Exosomes Involved in Female Pathologies

Several studies proposed exosomes as some of the protagonists involved in a high number of female pathologies—such as premature ovarian failure (POF), recurrent implantation failure (RIF), preeclampsia, endometriosis, cervical and endometrial cancer, and polycystic ovarian syndrome (PCOS)—delivering different cargoes, especially miRNAs, which could be regarded as diagnostic biomarkers [47,143,155,156,157,158] (Figure 2). The investigation of regulatory disorders caused by altered EV secretion, modifying the regulation of genes and protein expression and several signaling pathways such as wingless signaling pathway (WNT), transforming growth factor beta (TGF-β), neurotrophins, insulin signaling pathways, mitogen-activated protein kinase (MAPK), epidermal growth factor receptor (ErbB) pathways and pathways associated with ubiquitin, could offer insights into implantation failure in women with reproductive diseases [159,160]. For instance, a recent study conducted by Zhou et al. illustrates, for the first time, the differential expression patterns of exosomal miRNAs from endometrial stroma cells of women with endometriosis-associated infertility [143]. Hsa-miR-494-3p, hsa-miR-10b-3p, hsa-125b-2-3p, and hsa-miR-1343-3p display higher expression levels, and some of their predicted target genes are related to endometrial receptivity: homeobox A10 (*HOXA10*) and leukemia inhibitory factor (*LIF*). HOXA10 is involved in morphological arrangements of the uterus and in endometrial regeneration during the menstrual cycle [144,145]; LIF is one of the most significant cytokines, essential for regular implantation [146]. Their expression is significantly decreased in patients with endometriosis, affecting endometrial receptivity and implantation [143]. Another study revealed that circulating exosomes in PCOS follicular fluid had differential miRNA expression compared to healthy female controls: miR-146a-5p and miR-126-3p expression levels were increased, while miR-20b-5p, miR-106a-5p, and miR-18a-3p showed a decreasing trend in PCOS patients [147]. Computational analysis highlighted the involvement of miRNAs in the MAPK signaling pathway, axon guidance, circadian rhythms, endocytosis, and tumorigenesis circuits, suggesting that they may confer a risk of PCOS [147]. Li, H., et al., in a study conducted in 2020, isolated exosomes from women with preeclampsia, reporting that the concentration and mean diameter of plasma exosomes were greater than healthy controls (women with uncomplicated term pregnancies) [148]. The authors found the up-regulation of miR-153-3p and miR-325-3p into exosomes from preeclampsia [148], which are known to be able to inhibit cell proliferation and promote apoptosis [149]. Recently, researchers have started to investigate the relationship between exosomal miRNAs and their influence on ovarian cancer [158]. Previous studies have revealed that exosomes could influence chemo-susceptibility in recipient cells by regulating different biological pathways, including cell cycle and apoptosis: for instance, miR-106a, miR-130a, miR-221, miR-222, miR-433, and miR-591 are introduced as modulators of drug resistance in ovarian cancer [150,151]. Additionally, a newer analysis indicated that macrophage-derived exosomes transfer miR-223 to epithelial ovarian cancer cells to promote drug resistance through the PI3K/AKT signaling pathway [152,158]. Additionally, Li et al. [153] showed that cancer-associated fibroblast (CAF)-derived exosomes induce endometrial cancer progression, partially due to the loss of miR-148b in the exosomes, which represent an important tumor suppressor. MiR-148b targets DNA (cytosine-5) methyltransferase1 (*DNMT1*), which suppresses endometrial cancer metastasis by increasing epithelial–mesenchymal transition (EMT) [153]. Another study observed that exosomal miR-320a, derived from CAFs, has lower expression in endometrial cancer cells and tissues: it targets hypoxia-inducible factor 1 subunit alpha (*HIF1α*), which reduces vascular endothelial growth factor a (VEGFA) expression, inhibiting cell proliferation [154]. Further investigation into the central role of exosomes and their miRNAs cargo in the pathophysiology of reproduction is required to elucidate the effect of exosomes on the activity of cancer. Better approaches to learning their activities in the female reproductive system secretome could promote the development of innovative diagnostic and therapeutic tools [22,158].

## 5. Conclusions and Future Perspectives

This review summarizes the principal implications of exosomal vesicles in a wide range of biological processes related to reproduction, acting as essential mediators of intercellular communication and major regulators of cell functions. The great versatility of exosomes is evident in their involvement in many processes including germinal cell development and regulation of the reproductive system, tumor growth, tissue homeostasis, immune regulation, and disease progression. However, despite notable progress in recent years, several challenges remain to be addressed. Firstly, the standardization of isolation and characterization methods is necessary to discriminate the diverse subpopulations of EVs and compare different studies from different laboratories. Unfortunately, a large number of papers focus only on specific cargo molecules (e.g., microRNA, lncRNA or proteins); it would be useful to have an overall view of the molecules inside extracellular vesicles on the same sample. More needs to be known about the mechanisms that allow the correct cargo selection and recognition signals among vesicles and target cells. Cell culture functional experiments are able to verify miRNA roles, identifying the mRNAs regulated by a specific miRNA. However, even if studies on exosomes based on cell cultures could provide us some significant information (for example, embryo-derived exosomes up-taken by endometrial cells, the action of exosomes contrasting oxidative stress, etc.), they are surely not able to recapitulate EV functions in vivo due to the complex regulation mechanisms of gene expression. In the field of reproductive biology, in clinical and bio-technological research, these studies could improve pregnancy and live birth rates in IVF, as well as ensuring the optimization of exosome-based therapeutic strategies for clinical translation medicine. Finally, EV investigations in basic research could clarify the unknown points within complex pathways regulating the different steps of the reproductive process, the main prerequisite of living organisms, to ensure the continuation of the species.

## References

[1] Sung B.H., Parent C.A., Weaver A.M. (2021). Extracellular vesicles: Critical players during cell migration. Dev. Cell.

[2] Ginini L., Billan S., Fridman E., Gil Z. (2022). Insight into Extracellular Vesicle-Cell Communication: From Cell Recognition to In-tracellular Fate. Cells.

[3] Yáñez-Mó M., Siljander P.R.-M., Andreu Z., Bedina Zavec A., Borràs F.E., Buzas E.I., Buzas K., Casal E., Cappello F., Carvalho J. (2015). Biological properties of extracellular vesicles and their physiological functions. J. Extracell. Vesicles.

[4] Di Bella M.A. (2022). Overview and Update on Extracellular Vesicles: Considerations on Exosomes and Their Application in Modern Medicine. Biology.

[5] Muthu S., Bapat A., Jain R., Jeyaraman N., Jeyaraman M. (2021). Exosomal therapy—A new frontier in regenerative medicine. Stem Cell Investig..

[6] Dai J., Su Y., Zhong S., Cong L., Liu B., Yang J., Tao Y., He Z., Chen C., Jiang Y. (2020). Exosomes: Key players in cancer and potential therapeutic strategy. Signal Transduct. Target. Ther..

[7] Zhang K., Cheng K. (2023). Stem cell-derived exosome versus stem cell therapy. Nat. Prod. Res..

[8] Baruah H., Sarma A., Basak D., Das M. (2024). Exosome: From biology to drug delivery. Drug Deliv. Transl. Res..

[9] Willms E., Cabañas C., Mäger I., Wood M.J.A., Vader P. (2018). Extracellular Vesicle Heterogeneity: Subpopulations, Isolation Techniques, and Diverse Functions in Cancer Progression. Front. Immunol..

[10] Abramowicz A., Widłak P., Pietrowska M. (2019). Different Types of Cellular Stress Affect the Proteome Composition of Small Extracellular Vesicles: A Mini Review. Proteomes.

[11] Menck K., Bleckmann A., Schulz M., Ries L., Binder C. (2017). Isolation and Characterization of Microvesicles from Peripheral Blood. J. Vis. Exp..

[12] Yu L., Zhu G., Zhang Z., Yu Y., Zeng L., Xu Z., Weng J., Xia J., Li J., Pathak J.L. (2023). Apoptotic bodies: Bioactive treasure left behind by the dying cells with robust diagnostic and therapeutic application potentials. J. Nanobiotechnol..

[13] Rajput A., Varshney A., Bajaj R., Pokharkar V. (2022). Exosomes as New Generation Vehicles for Drug Delivery: Biomedical Ap-plications and Future Perspectives. Molecules.

[14] Andronico F., Battaglia R., Ragusa M., Barbagallo D., Purrello M., Di Pietro C. (2019). Extracellular Vesicles in Human Oogenesis and Implantation. Int. J. Mol. Sci..

[15] Xu M., Ji J., Jin D., Wu Y., Wu T., Lin R., Zhu S., Jiang F., Ji Y., Bao B. (2023). The biogenesis and secretion of exosomes and multivesicular bodies (MVBs): Intercellular shuttles and implications in human diseases. Genes Dis..

[16] Yim K.H.W., Krzyzaniak O., Al Hrout A., Peacock B., Chahwan R. (2023). Assessing Extracellular Vesicles in Human Biofluids Using Flow-Based Analyzers. Adv. Healthc. Mater..

[17] Calvani R., Picca A., Guerra F., Coelho-Junior H.J., Bucci C., Marzetti E. (2022). Circulating extracellular vesicles: Friends and foes in neurodegeneration. Neural Regen. Res..

[18] Hánělová K., Raudenská M., Masařík M., Balvan J. (2024). Protein cargo in extracellular vesicles as the key mediator in the progression of cancer. Cell Commun. Signal..

[19] Donoso-Quezada J., Ayala-Mar S., González-Valdez J. (2021). The role of lipids in exosome biology and intercellular communication: Function, analytics and applications. Traffic.

[20] Kim H., Jang H., Cho H., Choi J., Hwang K.Y., Choi Y., Kim S.H., Yang Y. (2021). Recent Advances in Exosome-Based Drug Delivery for Cancer Therapy. Cancers.

[21] Johnstone R.M., Adam M., Hammond J.R., Orr L., Turbide C. (1987). Vesicle formation during reticulocyte maturation. Association of plasma membrane activities with released vesicles (exosomes). J. Biol. Chem..

[22] Ghafourian M., Mahdavi R., Jonoush Z.A., Sadeghi M., Ghadiri N., Farzaneh M., Salehi A.M. (2022). The implications of exosomes in pregnancy: Emerging as new diagnostic markers and therapeutics targets. Cell Commun. Signal..

[23] Li C.-Y., Liu S.-P., Dai X.-F., Lan D.-F., Song T., Wang X.-Y., Kong Q.-H., Tan J., Zhang J.-D. (2023). The emerging role of exosomes in the development of testicular. Asian J. Androl..

[24] Russell A.E., Sneider A., Witwer K.W., Bergese P., Bhattacharyya S.N., Cocks A., Cocucci E., Erdbrügger U., Falcon-Perez J.M., Freeman D.W. (2019). Biological membranes in EV biogenesis, stability, uptake, and cargo transfer: An ISEV position paper arising from the ISEV membranes and EVs workshop. J. Extracell. Vesicles.

[25] Leonardi A.A., Battaglia R., Morganti D., Faro M.J.L., Fazio B., De Pascali C., Francioso L., Palazzo G., Mallardi A., Purrello M. (2021). A Novel Silicon Platform for Selective Isolation, Quantification, and Molecular Analysis of Small Extracellular Vesicles. Int. J. Nanomed..

[26] Thery C., Witwer K.W., Aikawa E., Alcaraz M.J., Anderson J.D., Andriantsitohaina R., Antoniou A., Arab T., Archer F., Atkin-Smith G.K. (2018). Minimal infor-mation for studies of extracellular vesicles 2018 (MISEV2018): A position statement of the International Society for Extracellular Vesicles and update of the MISEV2014 guidelines. J. Extracell. Vesicles.

[27] Welsh J.A., Goberdhan D.C.I., O’Driscoll L., Buzas E.I., Blenkiron C., Bussolati B., Cai H., Di Vizio D., Driedonks T.A.P., Erdbrugger U. (2024). Minimal information for studies of extracellular vesicles (MISEV2023): From basic to advanced ap-proaches. J. Extracell. Vesicles.

[28] Konoshenko M.Y., Lekchnov E.A., Vlassov A.V., Laktionov P.P. (2018). Isolation of Extracellular Vesicles: General Methodologies and Latest Trends. BioMed Res. Int..

[29] Reclusa P., Verstraelen P., Taverna S., Gunasekaran M., Pucci M., Pintelon I., Claes N., de Miguel-Pérez D., Alessandro R., Bals S. (2020). Improving extracellular vesicles visualization: From static to motion. Sci. Rep..

[30] Szatanek R., Baj-Krzyworzeka M., Zimoch J., Lekka M., Siedlar M., Baran J. (2017). The Methods of Choice for Extracellular Vesicles (EVs) Characterization. Int. J. Mol. Sci..

[31] Schageman J., Zeringer E., Li M., Barta T., Lea K., Gu J., Magdaleno S., Setterquist R., Vlassov A.V. (2013). The Complete Exosome Workflow Solution: From Isolation to Characterization of RNA Cargo. BioMed Res. Int..

[32] Jankovicova J., Secova P., Michalkova K., Antalikova J. (2020). Tetraspanins, More than Markers of Extracellular Vesicles in Repro-duction. Int. J. Mol. Sci..

[33] Zhang H., Lyden D. (2019). Asymmetric-flow field-flow fractionation technology for exomere and small extracellular vesicle sepa-ration and characterization. Nat. Protoc..

[34] Kalluri R., LeBleu V.S. (2020). The biology, function, and biomedical applications of exosomes. Science.

[35] Shao H., Im H., Castro C.M., Breakefield X., Weissleder R., Lee H. (2018). New Technologies for Analysis of Extracellular Vesicles. Chem. Rev..

[36] He J., Ren W., Wang W., Han W., Jiang L., Zhang D., Guo M. (2022). Exosomal targeting and its potential clinical application. Drug Deliv. Transl. Res..

[37] Hoshino A., Costa-Silva B., Shen T.L., Rodrigues G., Hashimoto A., Tesic Mark M., Molina H., Kohsaka S., Di Giannatale A., Ceder S. (2015). Tumour exosome integrins determine or-ganotropic metastasis. Nature.

[38] Kawamura S., Iinuma H., Wada K., Takahashi K., Minezaki S., Kainuma M., Shibuya M., Miura F., Sano K. (2019). Exo-some-encapsulated microRNA-4525, microRNA-451a and microRNA-21 in portal vein blood is a high-sensitive liquid biomarker for the selection of high-risk pancreatic ductal adenocarcinoma patients. J. Hepato-Biliary-Pancreat. Sci..

[39] Cai X., Janku F., Zhan Q., Fan J.-B. (2015). Accessing Genetic Information with Liquid Biopsies. Trends Genet..

[40] Valadi H., Ekström K., Bossios A., Sjöstrand M., Lee J.J., Lötvall J.O. (2007). Exosome-mediated transfer of mRNAs and microRNAs is a novel mechanism of genetic exchange between cells. Nat. Cell Biol..

[41] Qiu Y., Li P., Zhang Z., Wu M. (2021). Insights into Exosomal Non-Coding RNAs Sorting Mechanism and Clinical Application. Front. Oncol..

[42] Yu D., Li Y., Wang M., Gu J., Xu W., Cai H., Fang X., Zhang X. (2022). Exosomes as a new frontier of cancer liquid biopsy. Mol. Cancer.

[43] Irmer B., Chandrabalan S., Maas L., Bleckmann A., Menck K. (2023). Extracellular Vesicles in Liquid Biopsies as Biomarkers for Solid Tumors. Cancers.

[44] Li P., Kaslan M., Lee S.H., Yao J., Gao Z. (2017). Progress in Exosome Isolation Techniques. Theranostics.

[45] He C., Zheng S., Luo Y., Wang B. (2018). Exosome Theranostics: Biology and Translational Medicine. Theranostics.

[46] Kanwar S.S., Dunlay C.J., Simeone D.M., Nagrath S. (2014). Microfluidic device (ExoChip) for on-chip isolation, quantification and characterization of circulating exosomes. Lab A Chip.

[47] Kowalczyk A., Wrzecińska M., Czerniawska-Piątkowska E., Kupczyński R. (2022). Exosomes—Spectacular role in reproduction. Biomed. Pharmacother..

[48] Chen C., Zhang Z., Gu X., Sheng X., Xiao L., Wang X. (2023). Exosomes: New regulators of reproductive development. Mater. Today Bio.

[49] Machtinger R., Laurent L.C., Baccarelli A.A. (2016). Extracellular vesicles: Roles in gamete maturation, fertilization and embryo implantation. Hum. Reprod. Update.

[50] Qamar A.Y., Mahiddine F.Y., Bang S., Fang X., Shin S.T., Kim M.J., Cho J. (2020). Extracellular Vesicle Mediated Crosstalk Between the Gametes, Conceptus, and Female Reproductive Tract. Front. Vet. Sci..

[51] Capra E., Lange-Consiglio A. (2020). The Biological Function of Extracellular Vesicles during Fertilization, Early Embryo-Maternal Crosstalk and Their Involvement in Reproduction: Review and Overview. Biomolecules.

[52] Aleksejeva E., Zarovni N., Dissanayake K., Godakumara K., Vigano P., Fazeli A., Jaakma U., Salumets A. (2022). Extracellular vesicle research in reproductive science: Paving the way for clinical achievementsdagger. Biol. Reprod..

[53] Gervasi M.G., Visconti P.E. (2017). Molecular changes and signaling events occurring in spermatozoa during epididymal maturation. Andrology.

[54] Sullivan R., Saez F., Girouard J., Frenette G. (2005). Role of exosomes in sperm maturation during the transit along the male re-productive tract. Blood Cells Mol. Dis..

[55] Cheng C.Y., Mruk D.D. (2012). The Blood-Testis Barrier and Its Implications for Male Contraception. Pharmacol. Rev..

[56] Rimmer M.P., Gregory C.D., Mitchell R.T. (2021). The transformative impact of extracellular vesicles on developing sperm. Reprod. Fertil..

[57] Amiri N., Mohammadi P., Allahgholi A., Salek F., Amini E. (2023). The potential of sertoli cells (SCs) derived exosomes and its therapeutic efficacy in male reproductive disorders. Life Sci..

[58] Dacheux J.-L., Belleannée C., Guyonnet B., Labas V., Teixeira-Gomes A.-P., Ecroyd H., Druart X., Gatti J.-L., Dacheux F. (2012). The contribution of proteomics to understanding epididymal maturation of mammalian spermatozoa. Syst. Biol. Reprod. Med..

[59] Belleannée C., Calvo E., Caballero J., Sullivan R. (2013). Epididymosomes Convey Different Repertoires of MicroRNAs Throughout the Bovine Epididymis1. Biol. Reprod..

[60] Ronquist G., Brody I. (1985). The prostasome: Its secretion and function in man. Biochim. Biophys. Acta.

[61] Ronquist G., Brody I., Gottfries A., Stegmayr B. (1978). An Mg^2+^ and Ca^2+^-stimulated adenosine triphosphatase in human prostatic fluid: Part I. Andrologia.

[62] Ronquist K.G., Ronquist G., Carlsson L., Larsson A. (2009). Human prostasomes contain chromosomal DNA. Prostate.

[63] Yanagimachi R., Kamiguchi Y., Mikamo K., Suzuki F., Yanagimachi H. (1985). Maturation of spermatozoa in the epididymis of the Chinese hamster. Am. J. Anat..

[64] Sullivan R., Saez F. (2013). Epididymosomes, prostasomes, and liposomes: Their roles in mammalian male reproductive physiology. Reproduction.

[65] Vojtech L., Woo S., Hughes S., Levy C., Ballweber L., Sauteraud R.P., Strobl J., Westerberg K., Gottardo R., Tewari M. (2014). Exosomes in human semen carry a distinctive repertoire of small non-coding RNAs with potential regulatory functions. Nucleic Acids Res..

[66] Arienti G., Carlini E., Palmerini C. (1997). Fusion of Human Sperm to Prostasomes at Acidic pH. J. Membr. Biol..

[67] Arienti G., Carlini E., Nicolucci A., Cosmi E.V., Santi F., Palmerini C.A. (1999). The motility of human spermatozoa as influenced by prostasomes at various pH levels. Biol. Cell.

[68] Li Q., Li H., Liang J., Mei J., Cao Z., Zhang L., Luo J., Tang Y., Huang R., Xia H. (2021). Sertoli cell-derived exosomal MicroRNA-486-5p regulates differentiation of spermatogonial stem cell through PTEN in mice. J. Cell. Mol. Med..

[69] Guan R.-L., Song W.-P., Gu S.-J., Tan X.-H., Gu Y.-Y., Song W.-D., Zeng J.-Y., Xin Z.-C. (2022). Proteomic analysis and miRNA profiling of human testicular endothelial cell-derived exosomes: The potential effects on spermatogenesis. Asian J. Androl..

[70] Salas-Huetos A., James E.R., Aston K.I., Carrell D.T., Jenkins T.G., Yeste M. (2020). The role of miRNAs in male human repro-duction: A systematic review. Andrology.

[71] Da Broi M.G., Giorgi V.S.I., Wang F., Keefe D.L., Albertini D., Navarro P.A. (2018). Influence of follicular fluid and cumulus cells on oocyte quality: Clinical implications. J. Assist. Reprod. Genet..

[72] Eppig J.J., Chesnel F., Hirao Y., O’Brien M.J., Pendola F.L., Watanabe S., Wigglesworth K. (1997). Oocyte control of granulosa cell development: How and why. Hum. Reprod..

[73] Matzuk M.M., Burns K.H., Viveiros M.M., Eppig J.J. (2002). Intercellular Communication in the Mammalian Ovary: Oocytes Carry the Conversation. Science.

[74] Buccione R., Schroeder A.C., Eppig J.J. (1990). Interactions between Somatic Cells and Germ Cells Throughout Mammalian Oogenesis1. Biol. Reprod..

[75] Adashi E.Y. (1994). Endocrinology of the ovary. Hum. Reprod..

[76] Senbon S., Hirao Y., Miyano T. (2003). Interactions between the Oocyte and Surrounding Somatic Cells in Follicular Development: Lessons from In Vitro Culture. J. Reprod. Dev..

[77] Hamel M., Dufort I., Robert C., Gravel C., Leveille M.-C., Leader A., Sirard M.-A. (2008). Identification of differentially expressed markers in human follicular cells associated with competent oocytes. Hum. Reprod..

[78] Di Pietro C. (2016). Exosome-mediated communication in the ovarian follicle. J. Assist. Reprod. Genet..

[79] da Silveira J.C., Veeramachaneni D.R., Winger Q.A., Carnevale E.M., Bouma G.J. (2012). Cell-Secreted Vesicles in Equine Ovarian Follicular Fluid Contain miRNAs and Proteins: A Possible New Form of Cell Communication Within the Ovarian Follicle1. Biol. Reprod..

[80] Santonocito M., Vento M., Guglielmino M.R., Battaglia R., Wahlgren J., Ragusa M., Barbagallo D., Borzi P., Rizzari S., Maugeri M. (2014). Molecular characterization of exosomes and their mi-croRNA cargo in human follicular fluid: Bioinformatic analysis reveals that exosomal microRNAs control pathways involved in follicular maturation. Fertil. Steril..

[81] Hung W.-T., Hong X., Christenson L.K., McGinnis L.K. (2015). Extracellular Vesicles from Bovine Follicular Fluid Support Cumulus Expansion1. Biol. Reprod..

[82] da Silveira J.C., de Ávila A.C.F.C.M., Garrett H.L., Bruemmer J.E., Winger Q.A., Bouma G.J. (2018). Cell-secreted vesicles containing microRNAs as regulators of gamete maturation. J. Endocrinol..

[83] Diez-Fraile A., Lammens T., Tilleman K., Witkowski W., Verhasselt B., De Sutter P., Benoit Y., Espeel M., D’Herde K. (2014). Age-associated differential microRNA levels in human follicular fluid reveal pathways potentially determining fertility and success of in vitro fertilization. Hum. Fertil..

[84] Griffiths G.S., Galileo D.S., Aravindan R.G., Martin-DeLeon P.A. (2009). Clusterin facilitates exchange of glycosyl phosphatidyl-inositol-linked SPAM1 between reproductive luminal fluids and mouse and human sperm membranes. Biol. Reprod..

[85] Murdica V., Giacomini E., Alteri A., Bartolacci A., Cermisoni G.C., Zarovni N., Papaleo E., Montorsi F., Salonia A., Viganò P. (2019). Seminal plasma of men with severe asthenozoospermia contain exosomes that affect spermatozoa motility and capacitation. Fertil. Steril..

[86] Kharazi U., Badalzadeh R. (2020). A review on the stem cell therapy and an introduction to exosomes as a new tool in reproductive medicine. Reprod. Biol..

[87] Baskaran S., Panner Selvam M.K., Agarwal A. (2020). Exosomes of male reproduction. Adv. Clin. Chem..

[88] Homer H., Rice G.E., Salomon C. (2017). Review: Embryo- and endometrium-derived exosomes and their potential role in assisted reproductive treatments–liquid biopsies for endometrial receptivity. Placenta.

[89] Zhang S., Lin H., Kong S., Wang S., Wang H., Wang H., Armant D.R. (2013). Physiological and molecular determinants of embryo implantation. Mol. Asp. Med..

[90] Galliano D., Pellicer A. (2014). MicroRNA and implantation. Fertil. Steril..

[91] Cha J., Sun X., Dey S.K. (2012). Mechanisms of implantation: Strategies for successful pregnancy. Nat. Med..

[92] Fritz R., Jain C., Armant D.R. (2014). Cell signaling in trophoblast-uterine communication. Int. J. Dev. Biol..

[93] Macklon N.S., Geraedts J.P.M., Fauser B.C.J.M. (2002). Conception to ongoing pregnancy: The ‘black box’ of early pregnancy loss. Hum. Reprod. Updat..

[94] Zinaman M.J., Clegg E.D., Brown C.C., O’Connor J., Selevan S.G. (1996). Estimates of human fertility and pregnancy loss. Fertil. Steril..

[95] Chard T. (1991). 11 Frequency of implantation and early pregnancy loss in natural cycles. Bailliere’s Clin. Obstet. Gynaecol..

[96] Sui C., Liao Z., Bai J., Hu D., Yue J., Yang S. (2023). Current knowledge on the role of extracellular vesicles in endometrial receptivity. Eur. J. Med. Res..

[97] Atwood C.S., Meethal S.V. (2016). The spatiotemporal hormonal orchestration of human folliculogenesis, early embryogenesis and blastocyst implantation. Mol. Cell. Endocrinol..

[98] Aplin J.D., Stevens A. (2022). Use of ‘omics for endometrial timing: The cycle moves on. Hum. Reprod..

[99] Es-Haghi M., Godakumara K., Häling A., Lättekivi F., Lavrits A., Viil J., Andronowska A., Nafee T., James V., Jaakma Ü. (2019). Specific trophoblast transcripts transferred by extracellular vesicles affect gene expression in endometrial epithelial cells and may have a role in embryo-maternal crosstalk. Cell Commun. Signal..

[100] Hart A.R., Khan N.L.A., Godakumara K., Dissanayake K., Piibor J., Muhandiram S., Eapen S., Heath P.R., Fazeli A. (2022). The role of extracellular vesicles in endometrial receptivity and their potential in reproductive therapeutics and diagnosis. Reprod. Biol..

[101] Ng Y.H., Rome S., Jalabert A., Forterre A., Singh H., Hincks C.L., Salamonsen L.A. (2013). Endometrial Exosomes/Microvesicles in the Uterine Microenvironment: A New Paradigm for Embryo-Endometrial Cross Talk at Implantation. PLoS ONE.

[102] Beal J.R., Ma Q., Bagchi I.C., Bagchi M.K. (2023). Role of Endometrial Extracellular Vesicles in Mediating Cell-to-Cell Communication in the Uterus: A Review. Cells.

[103] Greening D.W., Nguyen H.P., Elgass K., Simpson R.J., Salamonsen L.A. (2016). Human Endometrial Exosomes Contain Hormone-Specific Cargo Modulating Trophoblast Adhesive Capacity: Insights into Endometrial-Embryo Interactions1. Biol. Reprod..

[104] DesRochers L.M., Bordeleau F., Reinhart-King C.A., Cerione R.A., Antonyak M.A. (2016). Microvesicles provide a mechanism for intercellular communication by embryonic stem cells during embryo implantation. Nat. Commun..

[105] Ma Q., Beal J.R., Bhurke A., Kannan A., Yu J., Taylor R.N., Bagchi I.C., Bagchi M.K. (2022). Extracellular vesicles secreted by human uterine stromal cells regulate decidualization, angiogenesis, and trophoblast differentiation. Proc. Natl. Acad. Sci. USA.

[106] Liu M., Chen X., Chang Q.-X., Hua R., Wei Y.-X., Huang L.-P., Liao Y.-X., Yue X.-J., Hu H.-Y., Sun F. (2020). Decidual small extracellular vesicles induce trophoblast invasion by upregulating N-cadherin. Reproduction.

[107] Das M., Kale V. (2020). Extracellular vesicles: Mediators of embryo-maternal crosstalk during pregnancy and a new weapon to fight against infertility. Eur. J. Cell Biol..

[108] Franasiak J.M., Alecsandru D., Forman E.J., Gemmell L.C., Goldberg J.M., Llarena N., Margolis C., Laven J., Schoenmakers S., Seli E. (2021). A review of the pathophysiology of recurrent implantation failure. Fertil. Steril..

[109] Sehring J., Beltsos A., Jeelani R. (2021). Human implantation: The complex interplay between endometrial receptivity, inflammation, and the microbiome. Placenta.

[110] Fan W., Qi Y., Wang Y., Yan H., Li X., Zhang Y. (2023). Messenger roles of extracellular vesicles during fertilization of gametes, development and implantation: Recent advances. Front. Cell Dev. Biol..

[111] Giacomini E., Alleva E., Fornelli G., Quartucci A., Privitera L., Vanni V.S., Viganò P. (2019). Embryonic extracellular vesicles as informers to the immune cells at the maternal–fetal interface. Clin. Exp. Immunol..

[112] Cho K., Kook H., Kang S., Lee J. (2020). Study of immune-tolerized cell lines and extracellular vesicles inductive environment promoting continuous expression and secretion of HLA-G from semiallograft immune tolerance during pregnancy. J. Extracell. Vesicles.

[113] Bulmer J.N., Morrison L., Longfellow M., Ritson A., Pace D. (1991). Granulated lymphocytes in human endometrium: Histochemical and immunohistochemical studies. Hum. Reprod..

[114] Quenby S., Bates M., Doig T., Brewster J., Lewis-Jones D., Johnson P., Vince G. (1999). Pre-implantation endometrial leukocytes in women with recurrent miscarriage. Hum. Reprod..

[115] Wu H.-M., Chen L.-H., Hsu L.-T., Lai C.-H. (2022). Immune Tolerance of Embryo Implantation and Pregnancy: The Role of Human Decidual Stromal Cell- and Embryonic-Derived Extracellular Vesicles. Int. J. Mol. Sci..

[116] Kaminski V.d.L., Ellwanger J.H., Chies J.A.B. (2019). Extracellular vesicles in host-pathogen interactions and immune regulation—Exosomes as emerging actors in the immunological theater of pregnancy. Heliyon.

[117] Czernek L., Düchler M. (2020). Exosomes as Messengers between Mother and Fetus in Pregnancy. Int. J. Mol. Sci..

[118] Nakamura K., Kusama K., Ideta A., Kimura K., Hori M., Imakawa K. (2019). Effects of miR-98 in intrauterine extracellular vesicles on maternal immune regulation during the peri-implantation period in cattle. Sci. Rep..

[119] Nakamura K., Kusama K., Hori M., Imakawa K. (2021). The effect of bta-miR-26b in intrauterine extracellular vesicles on maternal immune system during the implantation period. Biochem. Biophys. Res. Commun..

[120] Poh Q.H., Rai A., Salamonsen L.A., Greening D.W. (2023). Omics insights into extracellular vesicles in embryo implantation and their therapeutic utility. Proteomics.

[121] Pallinger E., Bognar Z., Bogdan A., Csabai T., Abraham H., Szekeres-Bartho J. (2018). PIBF+ extracellular vesicles from mouse embryos affect IL-10 production by CD8+ cells. Sci. Rep..

[122] Kovács F., Fekete N., Turiák L., Ács A., Kőhidai L., Buzás E.I., Pállinger É. (2019). Unravelling the Role of Trophoblastic-Derived Extracellular Vesicles in Regulatory T Cell Differentiation. Int. J. Mol. Sci..

[123] Rai A., Poh Q.H., Fatmous M., Fang H., Gurung S., Vollenhoven B., Salamonsen L.A., Greening D.W. (2021). Proteomic profiling of human uterine extracellular vesicles reveal dynamic regulation of key players of embryo implantation and fertility during menstrual cycle. Proteomics.

[124] Ouyang Y., Mouillet J.-F., Coyne C., Sadovsky Y. (2014). Review: Placenta-specific microRNAs in exosomes—Good things come in nano-packages. Placenta.

[125] Noguer-Dance M., Abu-Amero S., Al-Khtib M., Lefevre A., Coullin P., Moore G.E., Cavaille J. (2010). The primate-specific mi-croRNA gene cluster (C19MC) is imprinted in the placenta. Hum. Mol. Genet..

[126] Yuan Z., Sun X., Jiang D., Ding Y., Lu Z., Gong L., Liu H., Xie J. (2010). Origin and evolution of a placental-specific microRNA family in the human genome. BMC Evol. Biol..

[127] Donker R.B., Mouillet J.F., Chu T., Hubel C.A., Stolz D.B., Morelli A.E., Sadovsky Y. (2012). The expression profile of C19MC microRNAs in primary human trophoblast cells and exosomes. Mol. Hum. Reprod..

[128] Luo S.S., Ishibashi O., Ishikawa G., Ishikawa T., Katayama A., Mishima T., Takizawa T., Shigihara T., Goto T., Izumi A. (2009). Human villous trophoblasts express and secrete placenta-specific mi-croRNAs into maternal circulation via exosomes. Biol. Reprod..

[129] Yang C., Song G., Lim W. (2019). Effects of extracellular vesicles on placentation and pregnancy disorders. Reproduction.

[130] Kambe S., Yoshitake H., Yuge K., Ishida Y., Ali M., Takizawa T., Kuwata T., Ohkuchi A., Matsubara S., Suzuki M. (2014). Human Exosomal Placenta-Associated miR-517a-3p Modulates the Expression of PRKG1 mRNA in Jurkat Cells1. Biol. Reprod..

[131] Kurian N.K., Modi D. (2019). Extracellular vesicle mediated embryo-endometrial cross talk during implantation and in pregnancy. J. Assist. Reprod. Genet..

[132] Saadeldin I., Oh H.J., Lee B. (2015). Embryonic–maternal cross-talk via exosomes: Potential implications. Stem Cells Clon. Adv. Appl..

[133] Nguyen H.P., Simpson R.J., Salamonsen L.A., Greening D.W. (2016). Extracellular Vesicles in the Intrauterine Environment: Challenges and Potential Functions. Biol. Reprod..

[134] Chen K., Liang J., Qin T., Zhang Y., Chen X., Wang Z. (2022). The Role of Extracellular Vesicles in Embryo Implantation. Front. Endocrinol..

[135] Scott R.T., Upham K.M., Forman E.J., Zhao T., Treff N.R. (2013). Cleavage-stage biopsy significantly impairs human embryonic implantation potential while blastocyst biopsy does not: A randomized and paired clinical trial. Fertil. Steril..

[136] Giacomini E., Vago R., Sanchez A.M., Podini P., Zarovni N., Murdica V., Rizzo R., Bortolotti D., Candiani M., Viganò P. (2017). Secretome of in vitro cultured human embryos contains extracellular vesicles that are uptaken by the maternal side. Sci. Rep..

[137] Bhadarka H.K., Patel N.H., Patel N.H., Patel M., Patel K.B., Sodagar N.R., Phatak A.G., Patel J.S. (2017). Impact of embryo co-culture with cumulus cells on pregnancy & implantation rate in patients undergoing in vitro fertilization using donor oocyte. Indian J. Med. Res..

[138] Pallinger E., Bognar Z., Bodis J., Csabai T., Farkas N., Godony K., Varnagy A., Buzas E., Szekeres-Bartho J. (2017). A simple and rapid flow cytometry-based assay to identify a competent embryo prior to embryo transfer. Sci. Rep..

[139] Capalbo A., Ubaldi F.M., Cimadomo D., Noli L., Khalaf Y., Farcomeni A., Ilic D., Rienzi L. (2016). MicroRNAs in spent blastocyst culture medium are derived from trophectoderm cells and can be explored for human embryo reproductive competence as-sessment. Fertil. Steril..

[140] Rosenbluth E.M., Shelton D.N., Wells L.M., Sparks A.E., Van Voorhis B.J. (2014). Human embryos secrete microRNAs into culture media—A potential biomarker for implantation. Fertil. Steril..

[141] Abu-Halima M., Häusler S., Backes C., Fehlmann T., Staib C., Nestel S., Nazarenko I., Meese E., Keller A. (2017). Micro-ribonucleic acids and extracellular vesicles repertoire in the spent culture media is altered in women undergoing In Vitro Fertilization. Sci. Rep..

[142] Battaglia R., Palini S., Vento M.E., La Ferlita A., Faro M.J.L., Caroppo E., Borzì P., Falzone L., Barbagallo D., Ragusa M. (2019). Identification of extracellular vesicles and characterization of miRNA expression profiles in human blastocoel fluid. Sci. Rep..

[143] Zhou W., Lian Y., Jiang J., Wang L., Ren L., Li Y., Yan X., Chen Q. (2020). Differential expression of microRNA in exosomes derived from endometrial stromal cells of women with endometriosis-associated infertility. Reprod. Biomed. Online.

[144] Ekanayake D.L., Malopolska M.M., Schwarz T., Tuz R., Bartlewski P.M. (2022). The roles and expression of HOXA/Hoxa10 gene: A prospective marker of mammalian female fertility?. Reprod. Biol..

[145] Du H., Taylor H.S. (2015). The Role of Hox Genes in Female Reproductive Tract Development, Adult Function, and Fertility. Cold Spring Harb. Perspect. Med..

[146] Kimber S.J. (2005). Leukaemia inhibitory factor in implantation and uterine biology. Reproduction.

[147] Jiang X., Li J., Zhang B., Hu J., Ma J., Cui L., Chen Z.-J. (2021). Differential expression profile of plasma exosomal microRNAs in women with polycystic ovary syndrome. Fertil. Steril..

[148] Li H., Ouyang Y., Sadovsky E., Parks W.T., Chu T., Sadovsky Y. (2020). Unique microRNA Signals in Plasma Exosomes from Pregnancies Complicated by Preeclampsia. Hypertension.

[149] Zeng H.F., Yan S., Wu S.F. (2017). MicroRNA-153-3p suppress cell proliferation and invasion by targeting SNAI1 in melanoma. Biochem. Biophys. Res. Commun..

[150] Liang T., Guo Q., Li L., Cheng Y., Ren C., Zhang G. (2016). MicroRNA-433 inhibits migration and invasion of ovarian cancer cells via targeting Notch1. Neoplasma.

[151] Azmi A.S., Bao B., Sarkar F.H. (2013). Exosomes in cancer development, metastasis, and drug resistance: A comprehensive review. Cancer Metastasis Rev..

[152] Zhu X., Shen H., Yin X., Yang M., Wei H., Chen Q., Feng F., Liu Y., Xu W., Li Y. (2019). Macrophages derived exosomes deliver miR-223 to epithelial ovarian cancer cells to elicit a chemoresistant phenotype. J. Exp. Clin. Cancer Res..

[153] Li B., Lu W., Qu J., Ye L., Du G., Wan X. (2019). Loss of exosomal miR-148b from cancer-associated fibroblasts promotes endometrial cancer cell invasion and cancer metastasis. J. Cell. Physiol..

[154] Zhang N., Wang Y., Liu H., Shen W. (2020). Extracellular vesicle encapsulated microRNA-320a inhibits endometrial cancer by sup-pression of the HIF1alpha/VEGFA axis. Exp. Cell Res..

[155] Schjenken J.E., Panir K., Robertson S.A., Hull M.L. (2019). Exosome-mediated intracellular signalling impacts the development of endometriosis—New avenues for endometriosis research. Mol. Hum. Reprod..

[156] Wang L., Wang L., Wang R., Xu T., Wang J., Cui Z., Cheng F., Wang W., Yang X. (2024). Endometrial stem cell-derived exosomes repair cisplatin-induced premature ovarian failure via Hippo signaling pathway. Heliyon.

[157] Zhang X., Zhang R., Hao J., Huang X., Liu M., Lv M., Su C., Mu Y.L. (2022). miRNA-122-5p in POI ovarian-derived exosomes promotes granulosa cell apoptosis by regulating BCL9. Cancer Med..

[158] Esfandyari S., Elkafas H., Chugh R.M., Park H.S., Navarro A., Al-Hendy A. (2021). Exosomes as Biomarkers for Female Repro-ductive Diseases Diagnosis and Therapy. Int. J. Mol. Sci..

[159] Mobarak H., Rahbarghazi R., Lolicato F., Heidarpour M., Pashazadeh F., Nouri M., Mahdipour M. (2019). Evaluation of the asso-ciation between exosomal levels and female reproductive system and fertility outcome during aging: A systematic review protocol. Syst. Rev..

[160] Jiang N.-X., Li X.-L. (2021). The Complicated Effects of Extracellular Vesicles and Their Cargos on Embryo Implantation. Front. Endocrinol..

